# Comment on “Bone Regulates Glucose Metabolism as an Endocrine Organ through Osteocalcin”

**DOI:** 10.1155/2016/9724929

**Published:** 2016-09-20

**Authors:** Hitesh Verma, Rajeev Garg

**Affiliations:** ^1^IKG Punjab Technical University, Kapurthala 144603, India; ^2^Department of Pharmaceutics, ASBASJSM College of Pharmacy, Bela, Ropar 140111, India; ^3^Overseas R&D Centre, Overseas HealthCare Pvt. Ltd., Phillaur, Punjab 144410, India

While reading the insightful review of Shao et al. [1] on the effect of osteocalcin (OC) on glucose homeostasis, we came across a few lacunas which we want to fill up so as to generalise the osteocalcin concept. Firstly, Shao et al. reported that the concentration of uncarboxylated osteocalcin (ucOC) is controlled by decarboxylation of carboxylated osteocalcin (cOC), which is not true; it has been reported in the literature that during the bone resorption process initially a small fraction of intact OC is released in the acidic environment created by osteoclasts, which undergoes enzymatic proteolysis and subsequently excretion of OC fragments in urine. In the same research, researchers also prove that incubation of cOC in simulated osteoclastic lacuna medium (pH 4.8, 37°C) for 48 hours did not result in decarboxylation process and it requires heating of cOC at 110°C for 3 hours in HCl (50 mmol/L) to cause its decarboxylation [2]. Clearly, such conditions are not available in a physiological environment; therefore, the concept of decarboxylation from our point of view requires further investigation. Secondly, it is still a questionable fact that impairment in OC (any form) levels causes disturbance in glucose homeostasis because hyperglycemia, both* in vitro* [3] and clinically [4], is reported to cause impaired osteoblastic activity too. Therefore, whether OC imbalance causes impaired glucose homeostasis or vice versa is a matter of research. Thirdly, if as mentioned by Shao et al. ucOC is the only active form of OC, then supplementation of Vitamin K or various antiresorptive therapies (raloxifene, alendronate, and strontium) should theoretically increase risk of Type 2 Diabetes because they decrease the level of circulating OC (ucOC) but it is not the case [5–9]; rather, they are reported to decrease the risk of Type 2 Diabetes. Therefore, which form of osteocalcin is responsible for benefit in glucose homeostasis is not clear till now. Moreover, the presence of *γ*-glutamate carboxylase has been reported on osteoblasts [10], absence of which results in impaired glucose homeostasis, clearly indicating the protective role of Vitamin K (cofactor of *γ*-glutamate carboxylase) in maintaining glucose balance. Lastly, Shao et al. did not list the major difference in OC physiology in mice and humans, while extrapolating the benefits; for example, mice have three genes for OC while humans have only one gene and Vitamin D3 downregulates the OC expression in mice while it causes the opposite in humans [11, 12]. We appreciate the work of Shao et al., for their efforts to compile such a controversial topic, and we are looking forward to further research on this topic.
